# Effect of Stress Generated by Occlusal Cyclic Force on Class I Bulk-Fill Composite Restoration Microleakage

**DOI:** 10.1055/s-0041-1735433

**Published:** 2021-10-21

**Authors:** Apirat Ritthiti, Vanthana Sattabanasuk, Kavin Karunratanakul, Pisol Senawongse

**Affiliations:** 1 Department of Conservative Dentistry, Faculty of Dentistry, Srinakharinwirot University, Bangkok, Thailand; 2 Department of Operative Dentistry and Endodontic, Faculty of Dentistry, Mahidol University, Bangkok, Thailand; 3 Biomedical Engineering Research Unit, National Metal and Materials Technology Center, Bangkok, Thailand

**Keywords:** composite restoration, cyclic loading, finite element, microleakage, occlusal force, stress

## Abstract

**Objective** This study aimed to evaluate the effects of different types and restorative techniques of Class I composite restorations with a single loading force on stress distribution and cyclic loading force on microleakage formation.

**Materials and Methods** Class I cavities were prepared in premolars with 4 mm depth and divided into six groups of different restorations with: (1) Filtek Z250; (2) a 3-mm-thick layer of Filtek Bulk Fill Flowable Restoration and covered with Z250; (3) a 1.5-mm-thick layer of flowable composite and covered with Z250; (4) lining all cavity with flowable composite and restored with Z250; (5) Filtek Bulk Fill Posterior Restoration; and (6) lining all cavity with flowable composite and restored with bulk-fill composite. The specimens with and without cyclic occlusal loading were subjected to microleakage observation. In addition, six different models of Class I restorations corresponding to the microleakage study were generated. Finite element analysis (FEA) was used to identify the stress distribution under a single loading force.

**Statistical Analysis** Data were statistically analyzed by two-way analysis of variance and multiple comparison. The significance level set at 0.05.

**Results** Cavity lining or restoration with flowable composite underneath conventional composite reduced stress on composite resin based on FEA (groups 2 and 3). The cyclic stress on composite increased microleakage. Restoration with flowable composite underneath conventional composite reduced the microleakage in Class I restoration (groups 2, 3, and 4).

**Conclusion** The most effective cavity lining with a flowable composite underneath conventional composite restoration was stress reduction under loading force resulting in microleakage reduction.

## Introduction

Posterior composite restorations have become widely used in dentistry because of their high success rate and conservation-related properties. Therefore, a decrease in the success rate after 5 years has been reported to be caused by secondary caries. Otherwise, marginal fracture and bulk fracture could necessitate the replacement of posterior composite restorations in long-term observation. 1 2 3

Clinically, posterior teeth are subjected to occlusal force with cyclic loading during mastication. Restorative materials are subjected to such cyclic stress and undergo material fatigue. This fatigue may cause defects that ultimately lead to a fracture in the materials at a stress level lower than that required for a single load application. 4 5 6 7 A recent study reported the effect of a single stress of 50 N loading on a Class I composite restoration from a two-dimensional model of finite element analysis. It produced the highest stress of 62.75 MPa at the enamel cavosurface. The stresses at the adhesive layer and resin composite were 54.63 and 42.11 MPa, respectively. Additionally, the use of the flowable composite lining reduced the stress. 8 With the application of cyclic stress, this 50 N stress may induce failure at the tooth/resin composite interface. 9 Additionally, different resin composites with different compositions showed different fatigue strengths for the different materials. 10

The currently developed composite resin called “bulk-fill composite resin” is claimed to have the potential of bulk-filling down to a depth of 4 mm. 11 12 13 This material also has the advantage of shrinkage stress reduction 14 and low cuspal deflection because of low shrinkage stress. 15 The bulk-fill composite resin can be classified into two groups regarding the viscosity: flowable or low viscosity composite and regular or high viscosity composite. The term “packable composite” is used with the relatively high viscosity composite. As mentioned by Leprince and coworkers, the use of bulk-fill composite resin can reduce the working time and lead clinicians to feel comfortable. Therefore, the disadvantage of this material is its poor mechanical properties compared with those of conventional composites. It is not appropriate to use for restoration in areas with high occlusal loads. 16

Additionally, the trends of restorations with resin composite lined with a flowable composite are growing. 17 The manufacturer recommends restoring the cavity deep, down to 4 mm, with one layer of bulk-fill flowable composite. Therefore, the flowable composite should be covered with a regular composite because of its low surface hardness and low elastic modulus. 18 19 20 Filling the cavity with a thick layer of flowable composite underneath the regular composite is also a questionable approach with respect to the ability and durability under occlusal loading.

To date, there are no publications in which fatigue testing was performed in combination with a microleakage test. This combination may stimulate the clinical condition in which a restored tooth is fatigued, resulting in microleakage and ultimately restoration failure. The null hypothesis of this study was that there were no significant differences in the microleakage of Class I restorations with different composite types with different restorative techniques under cyclic occlusal force.

## Materials and Methods

This study was performed by using protocols approved by the Faculty of Dentistry/Faculty of Pharmacy, Mahidol University Institutional Review Board.

### Finite Element Analysis

**Fig. 1:**
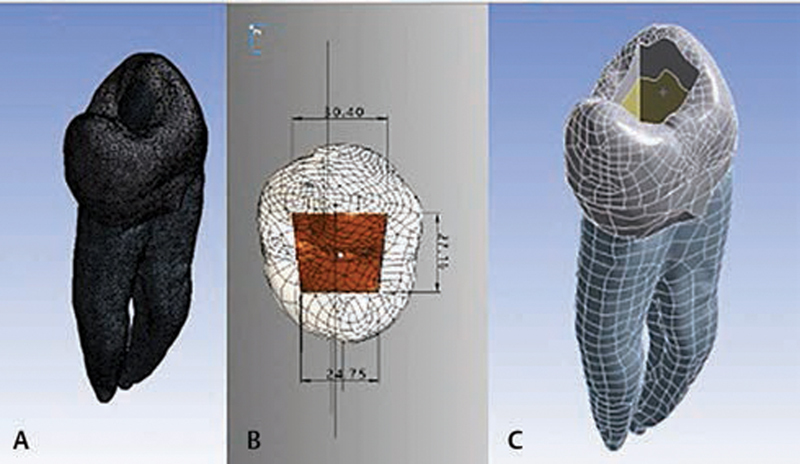
Tooth model with Class I cavity. ( **A** ) Three-dimensional tooth model, ( **B** ) dimensions of occlusal cavity are 30.40 × 27.16 × 24.74 × 27.16 mm, and ( **C** ) depth of the cavity is 40 mm from the central groove.

A three-dimensional image of a maxillary premolar tooth with a Class I preparation was taken by using a micro-CT (microcomputed tomography) scan (Skyscan 1173, Bruker, Kontich, Belgium). The micro-CT was set with a nominal voltage of 100 kV, current strength of 100 mm, and voxel size of 7.8 mm. The CT images were stored in DICOM format. Mimics software (Solidworks, Dassault Systems SolidWorks Corporation, Waltham, Massachusetts, United States) and power shape (Autodesk Inc, San Rafael, California, United States) were used to establish the tooth model with a Class I cavity for finite element analysis. The dimensions of the model are demonstrated in Fig. 1 .

The model consisted of enamel, dentin, a hybrid layer in dentin (assigned thickness = 1 μm), an adhesive layer (assigned thickness = 10 μm), and various methods of resin composite restoration. Six models were established according to the restorative techniques: Model 1: the cavity was restored with Filtek Z250 for a depth of 4 mm; Model 2: the bottom of the cavity was restored with Filtek Bulk Fill Flowable Restoration for 3 mm and covered occlusally with Filtek Z250 for 1 mm; Model 3: the bottom of the cavity was restored with Filtek Bulk Fill Flowable Restoration for 1.5 mm and covered occlusally with Filtek Z250 for 2.5 mm; Model 4: the prepared surfaces of the cavity were lined with 0.5 mm thick of Filtek Bulk Fill Flowable Restoration and the remaining cavity was restored with Filtek Z250; Model 5: the cavity was restored with Filtek Bulk Fill Posterior Restoration for a depth of 4 mm; and Model 6: the prepared surfaces of cavity were lined with a 0.5-mm-thick layer of Filtek Bulk Filtek Flowable Restoration and of remaining cavity was restored with Filtek Bulk Fill Posterior Restoration. Element types were tetrahedral with 563,365 elements and 840,399 nodes for Model 1; 584,804 elementsand 872,451 nodes for Model 2; 578,151elements and 871,546 nodes for Model 3; 570,659 elements and 871,546 nodes for Model 3; 570,659 elements and 870,439 nodes for Model 5; and 582,273 elements and 868,276 nodes for Model 6.

The tooth model was then imported into finite element analysis software (ANSYS Workbench, ANSYS Inc, Canonsburg, Pennsylvania, United States). To calculate the FEA, Poisson's ratio and the elastic modulus of all materials were entered ( Table 1 ). All materials were assumed to be isotropic and linearly elastic.

**Table 1:** Elastic modulus and Poisson's ratio of enamel, dentin, the hybrid layer, adhesive resin, flowable composites, and composite resin

Structures	Poisson's ratio	Elastic modulus (GPa)
Enamel	0.25 21	88.5 ± 5.43
Dentin	0.30 21	25.63 ± 3.23
Hybrid layer	0.30 22	12.75 ± 1.47
Filtek Z250	0.25 24	20.88 ± 1.26
Filtek Bulk Fill Posterior Restorative	0.25 24	11.60 ± 0.59
Filtek Bulk Fill Flowable Restorative	0.28 23	5.26 ± 0.05
Single Bond Universal	0.35 24	1.26 ± 0.51
Fuji II LC (as pulp)	0.35 23	8.23 ± 2.10

For the modulus of Filtek Z250, Filtek Bulk Fill Posterior Restorative, Filtek Bulk Fill Flowable Restorative, Single Bond Universal, and Fuji II LC, 10 extracted sound human premolars were used in this study. A Class I square butt-joint cavity was prepared with a water-cooled high-speed handpiece and a cylindrical diamond bur (FG 210/6, Intensive, Montagnola, Switzerland) by extending the cavity from the buccal cusp to the lingual cusp with a 2-mm buccolingual width and extending from the mesial to the distal pit with a 2.5-mm mesiodistal length. All cavities were prepared with a depth of 5 mm. The cavity was lined with a glass-ionomer cement (Fuji II LC) until achieving a depth of 3.5 mm from the cavity margin. The cavity was then finished with the diamond bur to obtain the cavity with dimension approximate of 4.0 × 2.5 × 3 mm (depth × width × length). The dimension of cavity was verified by the measurement with a periodontal probe by another investigator to ensure accuracy. The prepared cavities were divided into two groups of five teeth for restoration with (1) bonding with Single Bond Universal, Restoration with Filtek Bulk Fill Flowable Restoration for 3 mm and Filtek Z250 for 1 mm as shown in Model 2, and (2) bonding with Single Bond Universal, lining with a 0.5-mm thick layer of Filtek Bulk Fill Flowable Restoration and filling with Filtek Bulk Fill Posterior Restoration as shown in Model 6. The specimens were cured for 40 seconds incrementally with a light-emitting diode (LED) curing unit (Bluephase, Ivoclar Vivadent, Schaan, Liechtenstein) with the intensity of more than 1,100 mW/cm measured with a radiometer (Bluephase Meter, Ivoclar Vivadent, Schaan, Liechtenstein). The specimens of each group were sectioned buccolingually with a low-speed cutting machine (Isomet, Buehler, Lake Bluff, Illinois, United States) with a diamond blade (Isocut, Buehler, Lake Bluff, Illinois, United States). The cut specimens were then embedded in epoxy resin (Epon 815, Nissin, Tokyo, Japan) in polyvinylchloride rings. After the epoxy resin was set, the embedded specimens were polished with wet silicon carbide paper of decreasing abrasiveness (600; 800; 1,000; and 1,200 grit) and polished with diamond paste down to a grain size of 0.25 μm. The polished specimens were attached to a nanohardness testing system (NHT, CSM Instrument, Peseux, Switzerland) to measure the elastic modulus with 5,000 mgf loading on dentin, the adhesive and the resin composite and with 1,000 mgf loading on hybrid layers in a closed environment with dry conditions. Poisson's ratios used for the elastic modulus determination were included from previous studies. 21 22 23 24 Five indentations were performed in each substrate for each specimen. Moduli of elasticity (Gigapascal, GPa) were calculated from the attached computer.

**Fig. 2:**
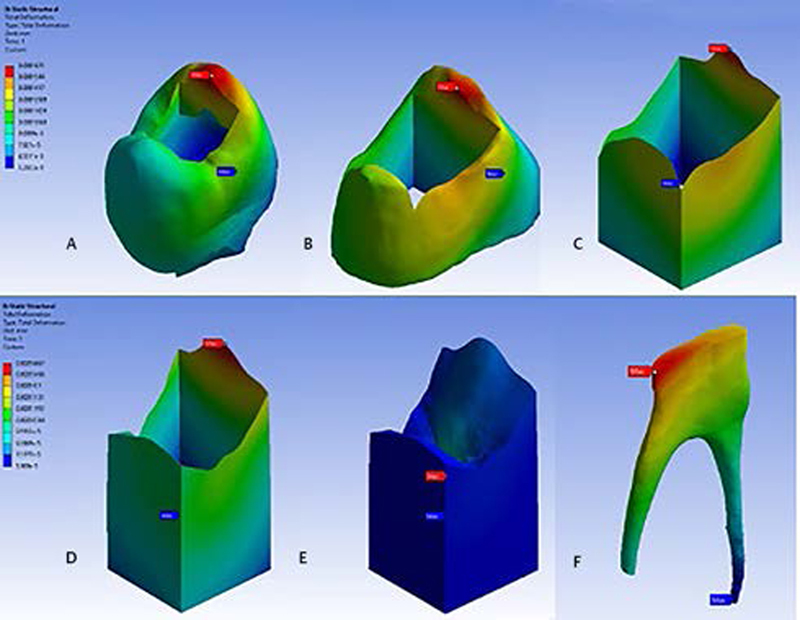
Stress distribution from finite element analysis of three-dimensional model 1 of each structure after receiving 50 N occlusal loading onto the composite resin. ( **A** ) enamel, ( **B** ) dentin, ( **C** ) hybrid layer, ( **D** ) adhesive layer, ( **E** ) composite resin, and ( **F** ) glass-ionomer cement.

**Fig. 3:**
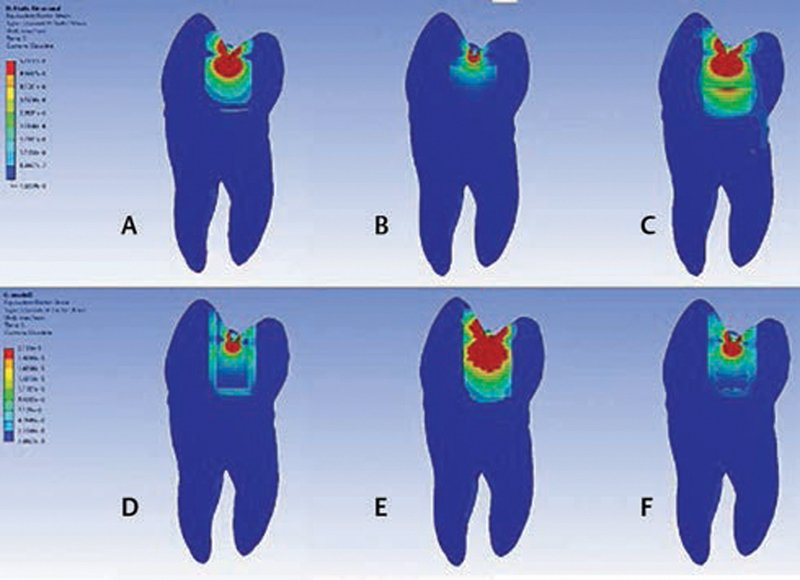
Finite element analysis. ( **A** ) Model 1, ( **B** ) Model 2, ( **C** ) Model 3, ( **D** ) Model 4, ( **E** ) Model 5, **(F),** and Model 6.

The models were subjected to an occlusal load of 50 N. 25 26 The load was applied at the reference point on the occlusal surface, parallel to the long axis. The representative finite element analysis of three-dimensional Model 1 of each structure after receiving a 50 N occlusal load onto the composite resin is shown in Fig. 2 . The Von Mises stress (MPa) and deformation (mm) from each structure were obtained from all models. The pictures from the finite element analysis are presented in Fig. 3 .

### Microleakage Testing with Microcomputed Tomography

Thirty maxillary second premolars of similar size without carious lesions or restorations extracted by orthodontic methods and kept in 0.1% thymol solution at 4°C were used within 6 months. A class I cavity was prepared with the high-speed cylindrical diamond bur by extending the cavity, following tooth preparation for nanoindentation testing. A new bur was used for every cavity preparation. Then, the cavities were divided into six groups of five teeth and restored as six models in the finite element analysis models. Before restorations, an adhesive (Single Bond Universal Adhesive) was applied to the cavities in self-etch mode following the manufacturer's instructions. Then, the specimens of Groups 1 to 6 were restored as mention in Model 1 to Model 6, sequently. Each increment of material was cured with the LED curing unit for 40 seconds. The materials used for restoration are demonstrated in Table 2 . All specimens were surface polished with three sequences of silicone-impregnated polishing devices (Astropol F/P/HP, Ivoclar Vivadent, Schaan, Liechtenstein) using a slow-speed handpiece running at 12,000 rpm under dry conditions immediately after polymerization. The restored teeth were kept in distilled water at 37°C for 24 hours. Then, microleakage testing with micro-CT was performed.

**Table 2:** Materials' details according to manufacturer's data

Materials	Compositions	Percent of filler by volume	Percent of filler by weight	Manufacturer
Filtek Z250	Resin matrix: Bis-GMA, TEGDMA, UDMA, Bis-EMA Filler: silica nanofillers, zirconia/silica nanocluster	60	78	3M ESPE, St. Paul, Minnesota, United States
Filtek Bulk Fill Posterior Restoration	Resin matrix: Bis-GMA, UDMA, Bis-EMA, AUDMA, AFM, DDDMA Filler: silica nanofillers, zirconia/silica nanocluster	58.4	76.5	3M ESPE, St. Paul, Minnesota, United States
Filtek Bulk Fill Flowable Restoration	Resin matrix: Bis-GMA, TEGDMA, UDMA, Bis-EMA, Bis-PMA Filler: silica nanofillers, zirconia/silica nanocluster	64.5	42.5	3M ESPE, St. Paul, Minnesota, United States
Single Bond Universal Adhesive	MDP phosphate monomer, dimethacrylate resins, HEMA, modified polyalkenoic acid copolymer, silane, filler, ethanol, water	n/a	n/a	3M ESPE, St. Paul, Minnesota, United States
GC Fuji II LC Capsule	Liquid: Distilled water, Polyacrylic acid, HEMA, UDMA, Comphorquinone Powder: Fluoroaluminosilicate glass			GC Corporation, Tokyo, Japan

Abbreviations: AFM, addition fragmentation monomer; AUDMA, aromatic urethane dimethacrylate; Bis-EMA, ethoxylated bisphenol A dimethacrylate; Bis-GMA, bisphenol A diglycidyl methacrylate; DDDMA, dodecanediol dimethacrylate; HEMA, hydroxyethyl methacrylate; MDP, methacryloyloxydecyl dihydrogen phosphate; n/a, not available; TEGDMA, triethyl glycol dimethacrylate; UDMA, urethane dimethacrylate.

After 24 hours of storage, the tooth surface of the specimen was coated with two layers of nail varnish leaving 1 mm between the interface of the tooth and the restoration. The coated specimens were immersed in 50% ammoniacal AgNO _3_ solution for 24 hours as a tracer, which developed in X-ray developing solution for 15 minutes and fixed for 2 minutes. Then, the specimens were subjected to micro-CT scanning. The micro-CT was set with a nominal voltage of 100 kV, current strength of 100 μA, and voxel size of 7.8 μm. Micro-CT images were used to investigate microleakage with CT analyzer software (CTAn 1.16, Skyscan, Kontich, Belgium). AgNO _3_ represents microleakage as radiopaque in micro-CT images. These micro-CT images were used as controls before cyclic loading.

**Fig. 4:**
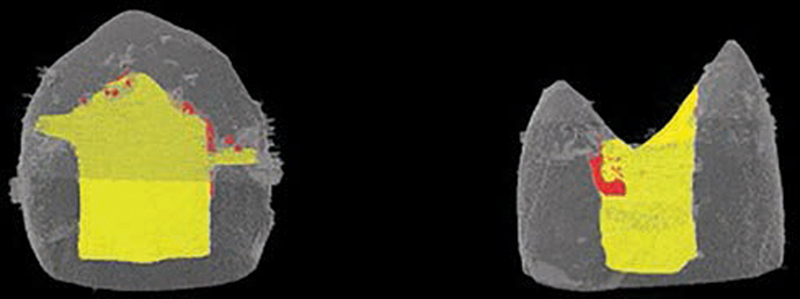
Microcomputed tomography images for microleakage analysis.

The specimens after micro-CT evaluation were further subjected to occlusal loading with 50 N force, 100,000 cycles, and 1.5 Hz frequency. Cyclic loading was performed under 100% humidity at the center of the restoration perpendicular to the tooth axis. Then, specimens were immersed in 50% ammoniacal AgNO _3_ solution for 24 hours as a tracer, which developed in X-ray developing solution for 15 minutes, fixed for 2 minutes, and subjected to microleakage evaluation with micro-CT. The microleakage data after cyclic loading were collected. The representative micro-CT images for microleakage analysis are presented in Fig. 4 . The red color areas demonstrate the microleakage.

### Statistical Analysis

The means and standard deviations of volumetric microleakage were calculated. The Shapiro–Wilk test was used for analysis of the distribution of data. The homogeneity of variances was analyzed by Levene's test. The influences of the restorative materials and placement techniques and the cyclic loading were analyzed with two-way ANOVA. Then, statistical analysis of volumetric microleakage before and after cyclic loading was analyzed by using paired sample *t* -tests. In addition, the difference among restorative materials and placement techniques within the same condition of cyclic loading was analyzed with one-way ANOVA and Tukey's HSD multiple comparison at 95% confidence intervals. SPSS version 18 (SPSS Inc., Chicago, Illinois, United States) was used for all statistical analyses.

## Results

The maximum von Mises stresses when an occlusal force (50 N) was applied to the central groove are presented in Table 3 . Occlusal loading created stress in the composite in all models. The highest stress in the composite was found in Model 1, in which the cavity was restored with only Filtek Z250 (11.616 MPa). The lowest stress in the composite was found in Model 2, in which the bottom of the cavity was restored with Filtek Bulk Fill Flowable Restoration for 3 mm and covered occlusally with Filtek Z250 for 1 mm (5.8669 MPa). The stress produced by occlusal loading for all models gradually decreased from high to low as follows: dentin, enamel, hybrid layer, and adhesive.

**Table 3:** Maximum Von Mises stresses (MPa) by the effect of occlusal loading in each substrate

Model	Enamel	Dentin	Hybrid layer	Adhesive	Flowable Co	Composite	Glass-ionomer cement
Model 1	0.10040	0.10673	0.06208	0.00979		11.616	0.01392
Model 2	0.17187	0.19882	0.06742	0.01779	0.22378	5.8669	0.01915
Model 3	0.12403	0.14739	0.05504	0.01054	0.02894	5.9837	0.01926
Model 4	0.10646	0.12683	0.04745	0.01056	0.01657	9.8271	0.02157
Model 5	0.12397	0.12989	0.05708	0.01210		6.6683	0.01505
Model 6	0.12658	0.15244	0.05553	0.01090	0.13162	9.5324	0.01909

The use of a flowable restoration composite could reduce the stress from occlusal loading in the composite. Therefore, stress occurring in enamel and dentin was clearly increased, especially when the flowable was used as in Model 2 (0.17187 MPa)

The deformation (mm) of each structure when an occlusal force (50 N) was applied to the central groove is presented in Table 4 . The maximum displacement was found in Model 6, in which the cavity wall was lined with Filtek Bulk Fill Flowable Restoration and restored with Filtek Bulk Fill Posterior Restoration (0.00165 mm). The lowest amount of deformation was found in Model 3, in which the bottom of the cavity was restored with Filtek Bulk Fill Flowable Restoration for 1.5 mm and covered occlusally with Filtek Z250 for 2.5 mm. The restoration with Filtek Bulk Fill Posterior Restoration (Groups 5 and 6) demonstrated a higher degree of deformation when compared with the restoration with Filtek Z250 for both lining and no lining groups.

**Table 4:** Deformation (mm) by the effect of occlusal loading in each substrate

Groups	Enamel	Dentin	Hybrid layer	Adhesive	Flowable Co	Composite	Glass-ionomer cement
Model 1	0.00016	0.00013	0.00013	0.00016		0.00084	0.00007
Model 2	0.00020	0.00015	0.00015	0.00020	0.00029	0.00087	0.00007
Model 3	0.00016	0.00012	0.00013	0.00016	0.00011	0.00078	0.00007
Model 4	0.00019	0.00014	0.00014	0.00019	0.00020	0.00102	0.00007
Model 5	0.00021	0.00016	0.00016	0.00021		0.00134	0.00008
Model 6	0.00024	0.00018	0.00018	0.00024	0.00024	0.00165	0.00009

According to two-way ANOVA, significant effect of the restorative materials and placement techniques ( *p* < 0.01) and the cyclic loading ( *p* < 0.01) to volumetric microleakage were found with the interaction between two factors ( *p* < 0.01). In addition, the power of test was investigated, which was 1.0. The effects of sample size were calculated that were 1.0 for the restorative materials and placement techniques, 1.0 for the cyclic loading and 0.99 for the interaction between two factors. The strong effect of sample size was found in this study.

The volumetric microleakage data for each group are shown in Table 5 . Only Group 6 (cavity lined with a 0.5-mm-thick layer of Filtek Bulk Fill Flowable Restoration and restored with Filtek Bulk Fill Posterior) showed no significant difference in microleakage before and after cyclic loading. Group 5 (cavity restored with Filtek Bulk Fill Posterior) showed the lowest volumetric microleakage both before and after cyclic loading. The highest volumetric microleakage was observed in Group 1 (restored with Filtek Z250) both before and after cyclic loading.

**Table 5:** Means and standard deviations of volumetric microleakage

Groups	Microleakage (mm ^3^ )	
	**Before cyclic loading**	**After cyclic loading**
Group 1	0.038 ± 0.012 ^aB^	0.096 ± 0.007 ^bD^
Group 2	0.015 ± 0.003 ^aA^	0.045 ± 0.007 ^bBC^
Group 3	0.023 ± 0.013 ^aAB^	0.057 ± 0.006 ^bC^
Group 4	0.014 ± 0.011 ^aA^	0.032 ± 0.110 ^bAB^
Group 5	0.014 ± 0.004 ^aA^	0.029 ± 0.008 ^bA^
Group 6	0.031 ± 0.015 ^aAB^	0.045 ± 0.010 ^aBC^

Note: Data with different uppercase letters in columns and lowercase letters in rows indicate significant differences ( *p* ≤0.05).

Thus, the lowest volumetric microleakage observed in Group 5 was not significantly different from that in Group 2 (the bottom of the cavity was restored with Filtek Bulk Fill Flowable Restoration for 3 mm and covered occlusally with Filtek Z250 for 1 mm); Group 3 (the bottom of the cavity was restored with Filtek Bulk Fill Flowable Restoration for 1.5 mm and covered occlusally with Filtek Z250 for 2.5 mm); Group 4 (the cavity was lined with a 0.5-mm thick layer of Filtek Bulk Fill Flowable Restoration and restored with Filtek Z250); and Group 6 (the cavity was lined with a 0.5-mm thick layer of Filtek Bulk Fill Flowable Restoration and restored with Filtek Bulk Fill Posterior Restoration) before cyclic loading conditions. No significant differences among Group 2, Group 4, and Group 6 were found after cyclic loading.

## Discussion

After the establishment of models, different restorative techniques and restorative resin-based materials were used in this study. Occlusal force was loaded onto composite restorations to mimic the clinical situation. The finite element analysis was able to show the pattern of stress distribution and provide stress detail within each structure in Class I composite restorations. 27 All models were simulations of a tooth-restoration system consisting of enamel, dentin, a hybrid layer, an adhesive layer, and resin composite. All components in the models were assumed to be isotropic, linearly elastic, and perfectly bounded together. Hence, the results for maximum Von Mises stress could not be interpreted by way of interfacial stress but only by stress consolidated in these models.

For this finite element analysis, six models were assigned to estimate the clinical use of bulk-fill composites compared with conventional hybrid composites (Filtek Z250). The bulk-fill composites could be divided according to consistency into two types: regular consistency (Filtek Bulk Fill Posterior Restoration) and low consistency (Filtek Bulk Fill Flowable Restoration). These two types of bulk-fill composites have different clinical applications. The regular consistency type can be used as the usual posterior composite. Moreover, its low consistency requires the occlusal surface to be protected with a conventional composite because of its poor mechanical properties. 18 28 29 In addition, a flowable material was used as a cavity lining in the model in an attempt to reduce the effect of occlusal stress 8 and improve adaptation. 28 30 Thus, two types of bulk-fill composites with different clinical applications were employed in the models.

In Model 1, the cavity was restored with Filtek Z250 to a depth of 4 mm and was designed to simulate normal posterior composite restoration. In Model 4, the prepared surface of the cavity was lined with a 0.5-mm thick layer of Filtek Bulk Fill Flowable Restoration, and the remaining cavity was restored with Filtek Z250 to simulate the use of a flowable composite as a stress absorbing layer in posterior restoration. 8

In Model 2, the bottom of the cavity was restored with Filtek Bulk Fill Flowable Restoration for 3 mm and covered occlusally with Filtek Z250 for 1 mm. In Model 3, the prepared surfaces of the cavity were lined with a 0.5-mm thick layer of Filtek Bulk Fill Flowable Restoration, and the remaining cavity was restored with Filtek Z250. This configuration was used to simulate the clinical application of flowable bulk-fill composites with different thicknesses according to the manufacturer's recommendation. 18 19 20

The application of bulk-fill composite resin with its regular consistency was used in Model 5 and Model 6. Model 5: the cavity restored with Filtek Bulk Fill Posterior Restoration for 4 mm depth was designed to simulate normal use of the bulk-fill composite. In addition, the flowable lining was applied to Model 6 to improve clinical adaptation. 28

The highest maximum Von Mises stress was found in Model 1 on the Filtek Z250 composite resin. The occlusal stress on Filtek Z250 could decrease with the application of low viscosity resin (flowable composite) underneath the hybrid composite resin (Model 2 and Model 3) and with the application of a flowable material as the lining (Model 4). The result was in agreement with that of a previous study. 8 The use of bulk-fill composite with regular consistency as a restorative material (Model 5 and Model 6) could reduce occlusal stress on the composite when compared with the models restored with Filtek Z250. The lower modulus of elasticity of the bulk-fill composite compared with the conventional hybrid composite ( Table 1 ) may affect the absorption and distribution of stress. 8 31 Since comparable mechanical properties of bulk-fill composites to conventional composites have been reported, 32 this may ensure the effective use of bulk-fill composites for posterior restoration. 29

The six models in finite element analysis were employed for the in vitro microleakage test of this study. Micro-CT was used to evaluate the microleakage with the use of a silver nitrate solution as a tracer. 33 The advantage of micro-CT is that it allows the volumetric leakage from the constructed three-dimensional model to be evaluated with a noninvasive process.

Various degrees of leakage were found in all groups and leakage was affected by cyclic loading of 50 N force with 100,000 cycles in this study. The use of universal adhesive with a self-etching mode onto enamel might have low efficiency for bonding with enamel because of self-etching mode was less effective, and lead to microleakage for all specimens. 34 To simulate 2 months of chewing, 100,000 cycles of cyclic loading were used. 35 Cyclic loading might initiate mechanical degradation, resulting in an increase in microleakage after cyclic loading in this study. 36 The leakage occurring before cyclic loading might act as a crack initiator. After progressive cyclic loading, microleakage might result from crack propagation. 37

In addition, without the cyclic loading, the microleakage was also found. The volumetric shrinkage of restorative materials may contribute for this observation. According to previous studies, the volumetric shrinkage of Z250, Filtek Bulk Fill Posterior Restoration and Filtek Bulk Fill Flowable Restoration were approximately 1.9, 1.1, and 1.4%. 38 39 The relatively high volumetric shrinkage in combination with high modulus of elasticity of Z250 might cause high shrinkage stress that affected high volumetric leakage found in Group 1. 14 40

The null hypothesis of this study was rejected. After cyclic loading, the capability of the flowable lining to reduce microleakage was found (Group 4 and Group 6). Therefore, the leakage of Group 4 was not significantly different from Group 5, in which the cavities were restored with bulk-fill composite. This might indicate that the greatest effectiveness is achieved when lining with a flowable material when a high modulus composite was used. This was comparable to the restoration by using only bulk-fill composite. The effective use of bulk-fill composites to reduce microleakage should be evident from their low polymerization shrinkage and relatively low elastic moduli that could reduce the stress effect, 14 38 resulting in low microleakage in this study.

Since this study performed in class I cavity with only enamel margin, the application to the cavity with dentin margin as found in class II should be concerned. The higher microleakage at dentin margin than at enamel margin has been reported. 41

## Conclusion

Within the limitations of this study, the use of low viscosity composite resin (flowable composite as a cavity lining or restoration underneath the conventional composite) can reduce the occlusal stress loading onto composite resin based on finite element analysis. The cyclic occlusal stress on the composite increased microleakage in Class I composite restorations. The most effective cavity lining with a flowable composite was found when this lining was used with a high elastic modulus composite resin.
